# Utility of Medical Legislation

**Published:** 1875-04

**Authors:** 


THE UTILITY OF MEDICAL LEGISLATION.— * * * In all our efforts to regulate the practice of medicine, we place ourselves in a very anomalous position towards the community. We take every pains and use every effort to protect the people against the evils of quackery, oftentimes against their will, and then shoulder ourselves with the responsibilities of enforcing the law afterwards. Evidently there is very little, if any, sympathy exercised towards us in such endeavors by the very people for whose benefits the law was originally framed. As far as we ourselves are concerned, the task is not only a thankless one, but is in its ultimate results so far from being satisfactory, that it tends to dishearten the most enthusiastic committee, or to render nugatory the best directed labors of the most faithful and conscientious board of examiners. * * *

The whole difficulty in making the different laws effective rests in a proper want of understanding on the part of the community. We can never expect to accomplish anything until we convince the people that these laws are intended more for their protection than ours. A learned profession should be above making any requests for legislative acts to benefit itself. If it cannot stand on its own merits, it has no claim whatever even to ordinary respect. Any effort to force such a consideration by a legal enactment is not only absurd, but positively ruinous. Our office is to protect the people as much as possible against quackery. If the community do not care to properly interpret these intentions, that is no concern of ours. Practically speaking, the people are not prepared to appreciate our motives, and will never be, until they are sufficiently educated to draw the line between quackery and legitimate medicine. When that time comes, there will be no difficulty in having suitable laws passed, and the people will themselves take the proper steps to punish any offenders.—*The Medical Record.*

READY FOR AN EMERGENCY.—According to the editor of the *Union Medicale,* a female practitioner in Paris was recently so overcome by the gush of blood in a case of *post-partum* hemorrhage that she fainted. By the time she recovered the patient was dead.—*Philadelphia Medical Times.*

"I am having myself taken in oil," said a well-known physician, complacently looking round. "Cod-liver, I suppose," growled a Jerroldian.—*London Society.*

